# Geometric consequences of electron delocalization for adenine tautomers in aqueous solution

**DOI:** 10.1007/s00894-014-2234-4

**Published:** 2014-05-15

**Authors:** Ewa D. Raczyńska, Mariusz Makowski

**Affiliations:** 1 Department of Chemistry, Warsaw University of Life Sciences (SGGW), ul. Nowoursynowska 159c, 02-776 Warszawa, Poland; 2 Laboratory of Intermolecular Interactions, Faculty of Chemistry, Univeristy of Gdańsk, ul. W. Stwosza 63, 80-308 Gdańsk, Poland

**Keywords:** Adenine, Electron delocalization, HOMED/Δ*E* relation, PCM//DFT, Solvent effects, Tautomers

## Abstract

Geometric consequences of electron delocalization were studied for all possible adenine tautomers in aqueous solution by means of *ab initio* methods {PCM(water)//DFT(B3LYP)/6-311+G(d,p)} and compared to those in the gas phase {DFT(B3LYP)/6-311+G(d,p)}. To measure the consequences of any type of resonance conjugation (π-π, n-π, and σ-π), the geometry-based harmonic oscillator model of electron delocalization (HOMED) index, recently extended to the isolated (DFT) and hydrated (PCM//DFT) molecules, was applied to the molecular fragments (imidazole, pyrimidine, 4-aminopyrimidine, and purine) and also to the whole tautomeric system. For individual tautomers, the resonance conjugations and consequently the bond lengths strongly depend on the position of the labile protons. The HOMED indices are larger for tautomers (or their fragments) possessing the labile proton(s) at the N rather than C atom. Solvent interactions with adenine tautomers slightly increase the resonance conjugations. Consequently, they slightly shorten the single bonds and lengthen the double bonds. When going from the gas phase to water solution, the HOMED indices increase (by less than 0.15 units). There is a good relation between the HOMED indices estimated in water solution and those in the gas phase for the neutral and ionized forms of adenine. Subtle effects, being a consequence of intramolecular interactions between the neighboring groups, are so strongly reduced by solvent that the relation between the HOMED indices and the relative energies for the neutral adenine tautomers seems to be better in water solution than in the gas phase.

**Electronic supplementary material:**

The online version of this article (doi:10.1007/s00894-014-2234-4) contains supplementary material, which is available to authorized users.

**Figure:**
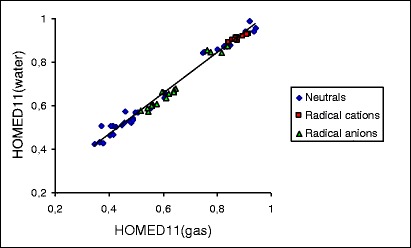
The total HOMED indices in water solution correlate well with those in the gas phase for the neutral and charged isomers of adenine

## Introduction

Various natural products, including pyrimidine and purine bases of nucleic acids, display prototropic tautomerism, the phenomenon which strongly influences their structure and biochemical transformations [1–4]. Depending on the number of the labile protons and on the number of tautomeric groups, they possess two or more tautomeric forms. Very often experimental techniques, applied to tautomeric compounds, cannot give the complete information on the structure of all possible tautomers. For example, application of spectroscopic methods such as ultraviolet (UV), infrared (IR), Raman, microwave (MW), nuclear magnetic resonance (NMR), mass spectrometry (MS), etc., leads mainly to the detection of the major tautomers, signals of which have significant intensities [1–3]. The minor and rare tautomers are not identified, probably because their amounts are too small and their signals cannot be distinguished from the background. Many internal and external factors such as polarity, aromaticity, stability of functionalities, acid–base properties of conjugated tautomeric sites, substituent and solvent effects, intra- and/or intermolecular interactions, oxidizing and reducing agents, ions, electrons, UV, γ-, and X-ray, etc. influence tautomeric equilibria. Depending on the tautomeric system, one or the other factor dictates the tautomeric preferences. In the case of simple tautomeric systems, a relation between prototropy and electron delocalization (also called resonance or mesomerism) has been signaled more than 50 years ago by Pauling [5]. This relation seems to be very simple, because prototropy by definition is associated with changes in electron delocalization [1–3]. However, electron delocalization (e.g., aromaticity) is not always the main factor that influences tautomeric equilibria and the relation between prototropy and electron delocalization is frequently perturbed by other factors [3].

**Chart 1:**
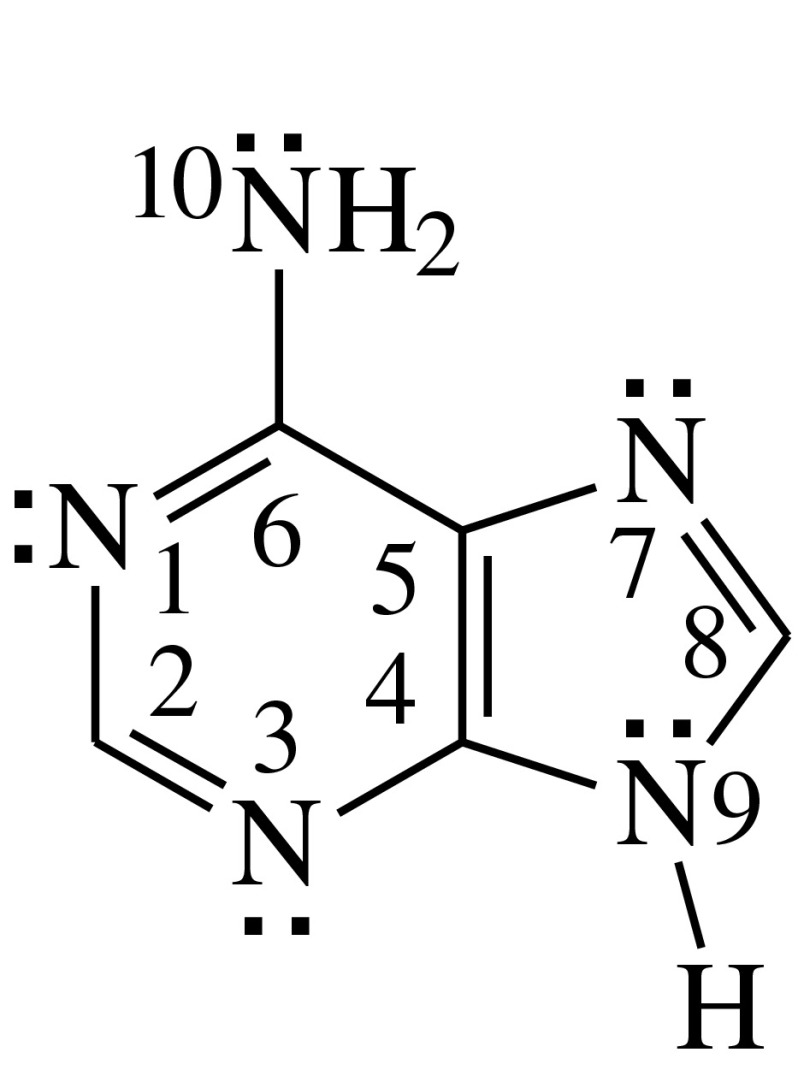
The canonical tautomer of adenine with all n- and π-electrons. The numbers refer to heavy atoms

In the case of adenine (Chart 1) – a well-known component part of nucleic acids, electron delocalization (aromaticity) seems to play a principal role for tautomeric conversions. To our knowledge, there are only a few reports discussing aromaticity for the canonical form [6–10] and for N-benzyl derivatives of three amine tautomers of adenine in the gas phase [11]. Lack of sufficient literature data encouraged us to study electron delocalization by means of geometric criterion for all possible prototropic tautomers of adenine in the gas phase using quantum-chemical methods [12]. A good relation between prototropy and electron delocalization, perturbed solely by some subtle internal effects, has been found. Since various processes for living organisms take place not only in nonpolar (lipids) but also in polar environments (enzymes, receptors, proteins, nucleoproteins, etc.) containing water molecules, it is interesting to study how electron delocalization varies in two extreme environments, that is, when proceeding from the gas phase (isolated molecules) to aqueous solution (hydrated molecules). For this reason, we extended our studies on electron delocalization to aqueous solution. We investigated the geometric consequences of electron delocalization for the hydrated adenine tautomers and compared them to those reported previously for isolated ones [12]. Such kind of studies gave the possibilities to estimate the extreme external effects. They should not be smaller than those for adenine included in DNA in living organisms systems.

To properly determine the distribution of π- and n-electrons for all individual tautomers of adenine, and to describe well the relation between prototropy and electron delocalization, the recently extended geometry-based harmonic oscillator model of electron delocalization (HOMED) procedure [13, 14] was applied to the geometries of the adenine isomers optimized in aqueous solution at the PCM(water)//DFT(B3LYP)/6-311+G(d,p) level [15]. The HOMED index is based on the original harmonic oscillator model of aromaticity (HOMA) idea [16, 17]. An abbreviation HOMED was proposed for the modified index [13], but it may also be abbreviated as moHOMA (modified original HOMA) or simply HOMA. Since the same phenomenon of electron delocalization takes place for the neutral, ionic and radical species, the same geometric criterion (HOMED) was applied for the neutral and ionized adenine isomers. It should be mentioned here that one can find in the literature many other indices of aromaticity which can be classified as energetic and magnetic ones [18–22]. For simple five-membered rings, good correlations occur between the geometry-based, energetic, and magnetic indices [21]. However, for polycyclic systems, the situation is more complex, because the magnetic indices are local descriptors and cannot be applied to the whole molecule, whereas the energetic indices are rather global ones, and cannot be used for the fragments. Solely the geometry-based indices can be applied for the whole molecule as well as for its fragments [18]. They can measure the geometric consequences of local and global electron delocalization for polycyclic systems.

The HOMA index, reformulated by Krygowski in 1993 (rHOMA) [23] and applied to heterocycles [18, 19], including the canonical structures of nucleobases [6], and the harmonic oscillator model of heterocyclic electron delocalization (HOMHED) index, proposed by Frizzo and Martins in 2012 [24] and based on the hypotheses of the HOMED index [14], were not applied here for the adenine tautomers. The reasons are as follows. In the rHOMA procedure, different measures of π-electron delocalization were employed for the reference CC, CX, and XY bonds [18, 19, 23], and the rHOMA index is inappropriate for the systems containing heteroatoms, e.g., purine [25]. It may be solely used for compounds possessing the same type of bonds, e.g., hydrocarbons [14, 26]. In the HOMHED procedure, the statistical reference bond lengths were applied [24]. The use of the statistical reference CC, CX, and XY bond lengths leads to some kind of statistical HOMHED values which do not describe well the real electron delocalization in heteroatomic systems.

## Methods

Geometries of adenine isomers in their ground states were fully optimized without symmetry constraints at the DFT(B3LYP)/6-311+G(d,p) level [27–30], and next re-optimized at the PCM(water)//DFT(B3LYP)/6-311+G(d,p) level [31, 32] as previously described [15]. For all calculations, the Gaussian 03 program [33] was used. The HOMED indices [13, 14] for the hydrated structures were estimated on the basis of the theoretically derived bond lengths (Tables S1 and S2, Supplementary material) according to Eq. (1) [16–18, 23] as described previously for purine [25]. In this equation, α is a normalization constant, *R*_o_ is the optimum bond length (assumed to be realized for fully delocalized system), *R*_i_ are the running bond lengths in the system, and *n* is the number of bonds taken into account. For the systems containing the even number of bonds (2i), the normalization α constants were calculated from Eq. (2), where *R*_s_ and *R*_d_ are the reference single and double bonds, respectively. This equation is similar to that proposed for the reformulated HOMA index [23]. In the case of the systems containing the odd number of bonds (2i + 1), i.e., (i + 1) single bonds and (i) double bonds, the normalization α constants were calculated from Eq. (3) [14]. The HOMED indices for the isolated (gas phase) adenine tautomers were taken from ref. [12].

1 $$ \mathrm{HOMED}=1-\left\{\upalpha \left(\mathrm{CC}\right)\cdot {{\displaystyle \sum \left[{R}_{\mathrm{o}}\left(\mathrm{CC}\right)-{R}_{\mathrm{i}}\left(\mathrm{CC}\right)\right]}}^2+\upalpha \left(\mathrm{CN}\right)\cdot {{\displaystyle \sum \left[{R}_{\mathrm{o}}\left(\mathrm{CN}\right)-{R}_{\mathrm{i}}\left(\mathrm{CN}\right)\right]}}^2\right\}/n $$

2 $$ \alpha =2\cdot {\left\{{\left({R}_{\mathrm{o}}-{R}_{\mathrm{s}}\right)}^2+{\left({R}_{\mathrm{o}}-{R}_{\mathrm{d}}\right)}^2\right\}}^{-1} $$

3 $$ \alpha =\left(2\mathrm{i}+1\right)\cdot {\left\{\left(\mathrm{i}+1\right)\cdot {\left({R}_{\mathrm{o}}-{R}_{\mathrm{s}}\right)}^2+\mathrm{i}\cdot {\left({R}_{\mathrm{o}}-{R}_{\mathrm{d}}\right)}^2\right\}}^{-1} $$

4 $$ {R}_{\mathrm{o}}=\left({R}_{\mathrm{s}}+\omega \cdot {R}_{\mathrm{d}}\right)/\left(1+\omega \right) $$

To quantitatively measure the geometric consequences of electron delocalization for the major, minor, and rare tautomers of adenine (A1-A23) in aqueous solution, the HOMED indices were estimated for the molecular fragments: imidazole (five bonds – HOMED5), pyrimidine (six bonds – HOMED6), 4-aminopyrimidine (seven bonds – HOMED7), and purine (ten bonds – HOMED10). They were also estimated for the whole tautomeric system including the exo−NH_2_/=NH group (11 bonds – HOMED11). For parametrization, the following *R*_s_, *R*_d_, and *R*_o_ values (in Å), calculated at the PCM(water)//DFT(B3LYP)/6-311+G(d,p) level [25], were taken here: 1.5308 (ethane), 1.3305 (ethene), and 1.3965 (benzene) for the CC bonds, and 1.4691 (methylamine), 1.2698 (methylimine), and 1.3354 (1,3,5-triazine) for the CN bonds, respectively. The *R*_o_ bond lengths, computed for benzene and 1,3,5-triazine (reference aromatic systems, HOMED = 1), are in accord with the harmonic oscillator method of optimization {Eq. (4)} [16, 17], in which ω (close to 2 for the CC and CN bonds) is the ratio of the stretching force constants for the pure double and single bonds. For the hydrated adenine fragments containing the even number of bonds, i.e., pyrimidine ring and purine system, the following normalization α constants, calculated according to Eq. (2), were used: 89.32 (CC) and 90.18 (CN). For those with the odd number of bonds, the normalization α constants were calculated according to Eq. (3). They are as follows: 79.59 (CC) and 80.34 (CN) for the imidazole fragment, 82.15 (CC) and 82.92 (CN) for the 4-aminopyrimidine fragment, and 84.64 (CC) and 85.42 (CN) for the whole tautomeric adenine system.

## Results and discussion

### Adenine tautomers

The tautomeric mixture of adenine contains 23 tautomers (Table 1), nine amine forms (**A1**-**A9**), and 14 imine forms (**A10**-**A23**) with the exo amine − NH_2_ and imine = NH group, respectively. When geometric isomerism of the imine forms is taken into account, 37 isomers are possible (Fig. S1, Supplementary material) [15]. The number of the prototropic tautomers for adenine is a consequence of the number of the labile protons (two) and conjugated tautomeric sites (ten). In the majority of theoretical documents, the amine-imine tautomeric conversions {−NH − C(R) = N − → − N = C(R) − NH−} have been investigated, and a maximum number of eight tautomers (**A1**, **A3**, **A7**, **A9**, **A13**,** A15**, **A18**, and **A20**) have been considered [34–38]. Application of NMR techniques have led to identification of three major tautomers (**A3**, **A7**, and **A9**) [39]. The enamine-imine conversions {>C = C(R) − NH − → > CH − C(R) = N−} have usually been omitted. Some exceptions are the anionic states of adenine for which the rare tautomers dominate [40, 41]. These variations of the tautomeric preferences and the hypothesis of rare tautomers, suggested by Watson and Crick [42] in 1953 for DNA mutations, encouraged us to study all 37 isomers for adenine [15].

**Table 1:** All possible 23 prototropic tautomers of adenine [15]

Tautomer	Position of H	Tautomer	Position of H	Tautomer	Position of H
**A1**	N1N10	**A9**	N9N10	**A17**	N3C5
**A2**	C2N10	**A10**	N1C2	**A18**	N3N7
**A3**	N3N10	**A11**	N1C4	**A19**	N3C8
**A4**	C4N10	**A12**	N1C5	**A20**	N3N9
**A5**	C5N10	**A13**	N1N7	**A21**	C5N7
**A6**	C6N10	**A14**	N1C8	**A22**	C5N9
**A7**	N7N10	**A15**	N1N9	**A23**	C8N9
**A8**	C8N10	**A16**	N3C4		

Adenine contains eight π- and ten n-electrons (Chart 1) which participate in electron delocalization during tautomeric conversions. More than one satisfactory Lewis structure, called resonance structures, can be drawn for each isomer [15, 43]. A single structure is composed of alternating single and double bonds. Electron delocalization for the resonance hybrid containing two or more resonance structures reveals shortening of the single bonds and lengthening of the double bonds. Solely full symmetry of the molecule leads to equalization of the corresponding bond lengths. Generally, the bond length alternation, or its lack of, is a consequence of various types of resonance conjugation in the molecule such as σ-π hyperconjugation, π-π and n-π conjugations. For the NH-NH tautomers of adenine, containing the labile protons at the N atoms, the π-π and n-π resonance conjugations are possible. For the NH-CH tautomers, possessing the labile protons at the N and C atoms, the σ-π hyperconjugation should be additionally considered. These different types of resonance conjugation lead to different geometric consequences of electron delocalization for the adenine tautomers. The number of the resonance structures substantially depends on the positions of the labile protons and on the positions of the double bonds in the Lewis structures. The number of the resonance structures affects the bond lengths, the stability of the tautomeric form, and its contribution in the tautomeric mixture. Consequently, prototropy and electron delocalization influence the structure of adenine, its physicochemical, chemical, and biochemical properties.

In this paper, all possible 37 isomers were considered for neutral adenine at the PCM(water)//DFT(B3LYP)/6-311+G(d,p) level. For selected isomers, the positively and negatively ionized forms (radical cations and anions) were then theoretically derived by removing and adding one election to the neutral isomer. These transformations refer to one-electron oxidation (**A** – e → **A** ^+•^) and one-electron reduction (**A** + e → **A** ^-•^). The neutral and ionized isomers considered here are geometrically stable. The CC and CN bond lengths (Tables S1 and S2, Supplementary material) calculated for the hydrated isomers were used to estimate the partial (HOMED5, HOMED6, HOMED7, and HOMED10) and total (HOMED11) geometry-based indices (Tables S3 and S4, Supplementary material). Generally, the partial HOMED values are larger for the fragments with the labile proton(s) at the N atom(s) than for those containing the C-sp^3^ atom with the labile proton. In most cases, the values of HOMED11 are between those of HOMED5 and HOMED7. Moreover, the values of HOMED11 are larger than those of HOMED10. The exo amino/imino group participates in the n-π or π-π conjugation increasing the number of possible resonance structures for the adenine system. The values of the relative energies (Δ*E*), calculated at the same level of theory in water solution, were taken from ref. [15]. They measure the relative stabilities of the hydrated adenine isomers. To study the solvent effects, the geometric and energetic data estimated for the hydrated isomers were compared with those previously reported for the isolated (gas phase) ones [12, 15].

### Solvent effects for the canonical and favored tautomers

**Chart 2:**
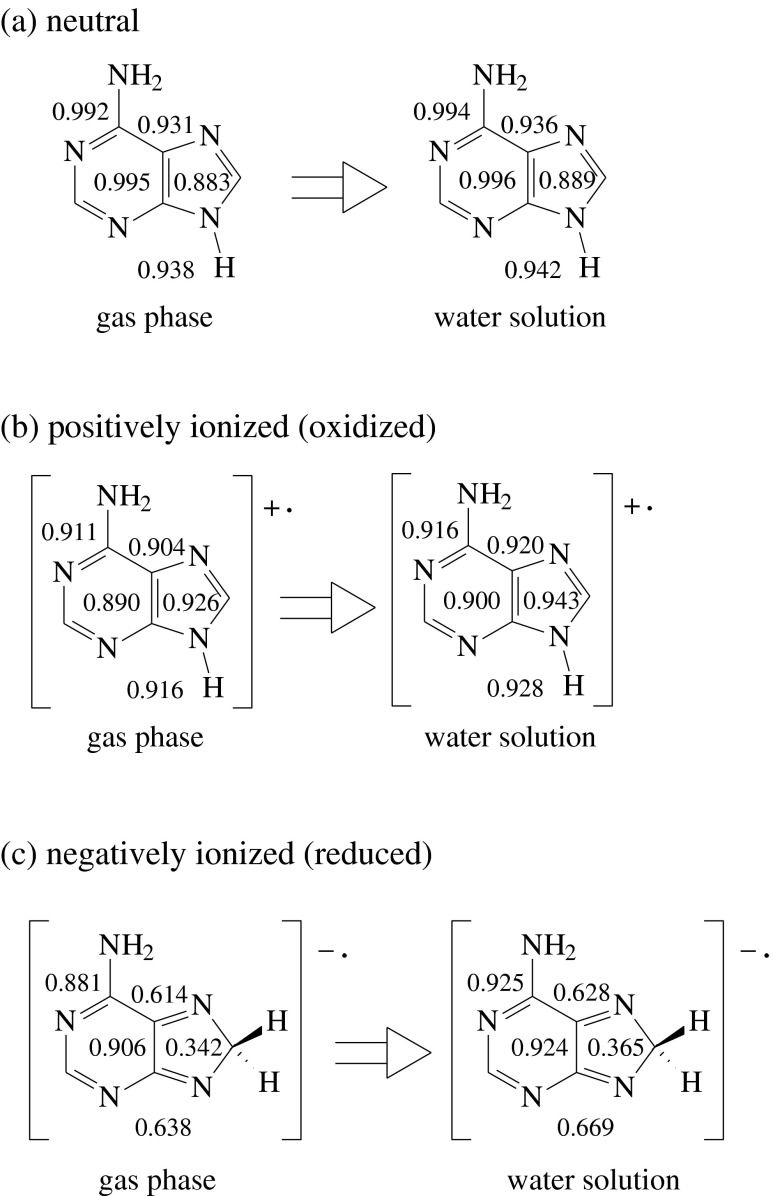
Variations of the partial and total HOMED indices for the favored tautomers of neutral (**a**), positively (**b**), and negatively (**c**) ionized adenine when proceeding from the gas phase to aqueous solution

The canonical tautomer **A9** is the favored form of neutral adenine in the gas phase and in aqueous solution [15, 34–41, 44–66]. Containing ten labile electrons in the purine system, **A9** satisfies the 4n + 2 rule, and it is aromatic [6–12]. The individual rings (imidazole and pyrimidine) contain six labile electrons and they also satisfy the 4n + 2 aromatic rule. The HOMED indices, estimated for the five (imidazole), six (pyrimidine), seven (4-aminopyrimidine), ten (purine), and 11 bonds (adenine), describe well the geometric consequences of electron delocalization and confirm the aromatic character of **A9** (Chart 2a) well. For hydrated **A9**, the HOMED5, HOMED6, HOMED7, HOMED10, and HOMED11 values (0.889, 0.996, 0.994, 0.936, and 0.942, respectively) are not very different from those found previously for isolated (gas phase) **A9** (0.883, 0.995, 0.992, 0.931, and 0.938, respectively [12]). When going from the gas phase to aqueous solution, the partial and total HOMED values slightly increase (by 0.001-0.006 units) indicating an increase of electron delocalization. The lowest solvent effect (0.001) takes place for the pyrimidine fragment which is already well delocalized (HOMED6 close to unity), and the largest one (0.006) for the imidazole fragment which is less delocalized (HOMED5 close to 0.9).

**Fig. 1:**
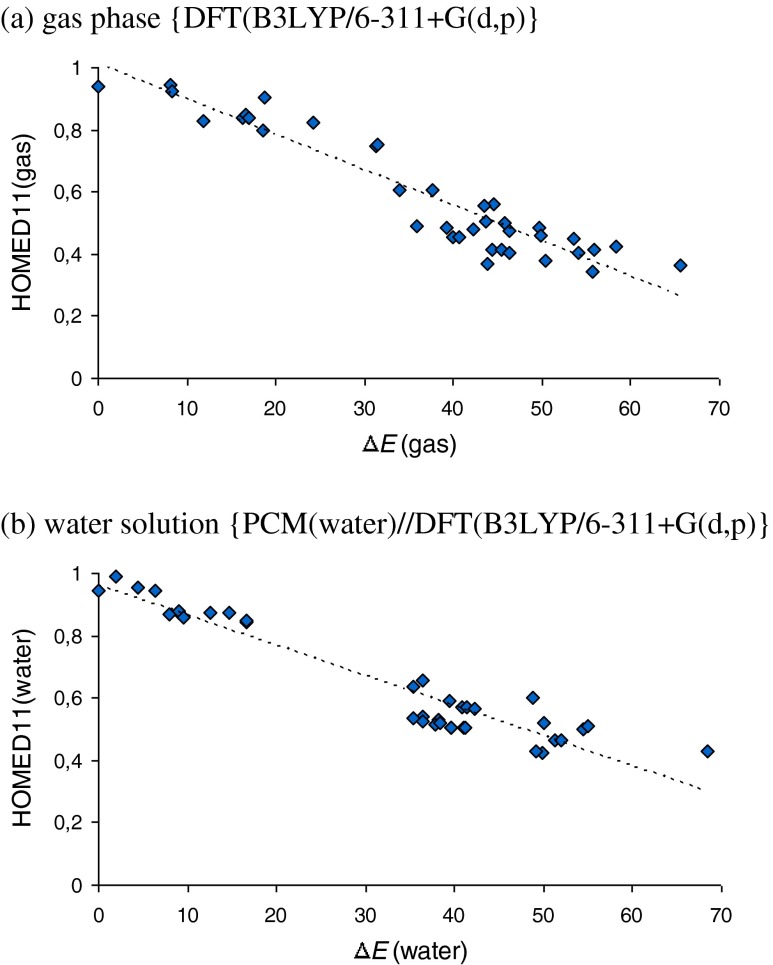
Comparison of correlations between the total HOMED values and the relative energies (Δ*E* in kcal mol^−1^) estimated for the neutral isomers of adenine in gas phase (**a**) and in water solution (**b**)

**Fig. 2:**
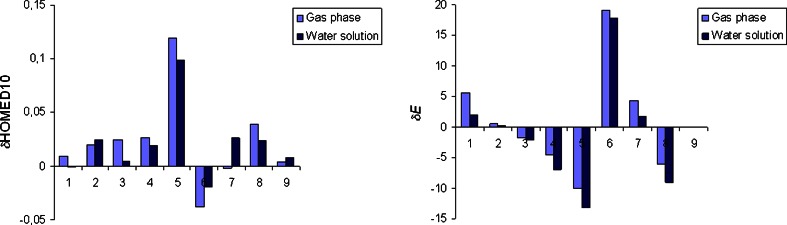
Variations of the geometric (δHOMED10) and energetic (δ*E* in kcal mol^−1^) total effects of the exo NH_2_ group for the neutral amine tautomers of adenine in the gas phase and in water solution. Numbers 1–9 correspond to the amine tautomers **A1**-**A9**, respectively

In the presence of oxidizing or reducing agents, adenine may lose or gain one electron and the charged radicals can be formed. They may also be generated electrochemically, photochemically, or during positive or negative ionization in various types of mass spectrometers. One-electron loss and one-electron gain affect the stability of adenine tautomers and their electron delocalization in different way. However, when proceeding from the gas phase to aqueous solution, the estimated partial and total HOMED values change in a similar way as those for the neutral form. For the positively ionized canonical form **A9** ^+•^, the HOMED5, HOMED6, HOMED7, HOMED10, and HOMED11 values estimated in water solution (0.943, 0.900, 0.916, 0.920, and 0.928, respectively, Chart 2b) are only slightly larger (by 0.005-0.017 units) than those found for **A9** ^+•^ in the gas phase (0.926, 0.890, 0.911, 0.904, and 0.916, respectively [12]).

The canonical tautomer is the favored form of positively ionized adenine [15]. In both environments (for isolated and hydrated molecule), the most important amount of the spin density exists on the N10 atom [15]. This means that one non-bonding electron is taken from the exo−NH_2_ group of the 4-aminopyrimidine fragment, rather than from the endo NH group of the imidazole ring. Lack of one electron on the N10 atom reduces the n-π conjugation of the exo−NH_2_ group with the pyrimidine ring, and decreases the corresponding HOMED values for **A9** ^+•^ in comparison to those for **A9**: HOMED6 (from 0.995 to 0.890 in the gas phase and from 0.996 to 0.900 in water solution) and HOMED7 (from 0.992 to 0.911 in the gas phase and from 0.994 to 0.916 in water solution). Consequently, the other HOMED values also decrease: HOMED10 for the purine fragment (from 0.931 to 0.904 in the gas phase and from 0.936 to 0.920 in water solution) and HOMED11 for the whole tautomeric adenine system (from 0.938 to 0.916 in the gas phase and from 0.942 to 0.928 in water solution). The decrease effects of the HOMED values in water solution (0.096, 0.078, 0.016, and 0.014 units, respectively) are only slightly lower than those observed in the gas phase (0.105, 0.081, 0.027, and 0.022 units, respectively [12]). One exception is the HOMED5 index, which value increases when going from **A9** to **A9** ^+•^ (from 0.883 to 0.926 in the gas phase and from 0.889 to 0.943 in water solution). The increase effects (0.043 and 0.054 units, respectively) confirm the fact that one non-bonding electron is not taken from the endo N atom of the imidazole fragment. The explanation is as follows. For the favored NH tautomer of isolated imidazole, for which one non-bonding electron may be taken from the endo NH group, positive ionization decreases the HOMED5 value for both the isolated (from 0.902 to 0.877) and hydrated tautomers (from 0.917 to 0.907) [67]. This effect is completely different than that for the adenine imidazole fragment.

Negative ionization reduces the stability of the canonical tautomer such that **A9** ^-•^ has larger energy value than **A8** ^-•^ − the NH-CH one, favored for reduced but considered as the rare form for neutral and oxidized adenine [15, 40, 41]. For **A8** ^-•^, the HOMED5, HOMED6, HOMED7, HOMED10, and HOMED11 values in water solution ( 0.365, 0.924, 0.925, 0.628, and 0.669, respectively, Chart 2c) are only slightly larger (by 0.014-0.044 units) than those found for **A8** ^-•^ in the gas phase (0.342, 0.906, 0.881, 0.614, and 0.638, respectively [12]). This form is non-aromatic due to the presence of the C8-sp^3^ atom in the imidazole fragment. Solely the pyrimidine and 4-aminopyrimidine fragments in **A8** ^-•^ are well delocalized (HOMEDs close to 0.9). The change of the tautomeric preference for negatively ionized adenine suggests that in this case aromaticity is not the main factor that affects the composition of the tautomeric mixture. This seems to be independent of environment.

### HOMED(water)/HOMED(gas) relation

First perusal of the total HOMED values estimated for the selected neutral and ionized forms of adenine in the gas phase and in water solution suggests that electron delocalization and consequently bond length alternation or its lack seem to be less sensitive to solvation than to electron transfer from and to adenine molecule (Table S3, Supplementary material). However, the estimated HOMED values are consistent with a general tendency of the resonance conjugation: more delocalized system, larger HOMED index value [14]. The HOMED indices are between zero and unity for the neutral and ionized isomers. Generally, the HOMED values increase for the adenine tautomers when proceeding from the gas phase to water solution. This increase may be explained by specific interactions of polar solvent with polar functional groups of adenine. Specific interactions (e.g., H-bonding) augment the geometry-based indices [3, 6]. For the neutral adenine forms, solvent effects seem to be larger for the NH-CH tautomers than for the NH-NH ones. The total HOMED indices increase by 0.011-0.136 and 0.004-0.097 units, respectively. Positive and negative ionization change the total HOMED indices in different way, in some cases even by more than 0.1 units. However, solvent effects seem to be similar to those for neutral tautomers: less delocalized tautomer larger solvent effect.

The parallelism of the geometric consequences of electron delocalization for the adenine tautomers in apolar (gas phase) and polar (water) environments leads to a good linear relation between the HOMED11 indices estimated for the whole tautomeric system (11 bonds) in the gas phase and in water solution (Fig. S2, Supplementary material). The HOMED11 values for the adenine tautomers in the gas phase were taken from ref. [12]. The HOMED(water)/HOMED(gas) relation seems to be independent of the oxidation state. It is common for the neutral and ionized isomers of adenine. Similarly, a good linear relation exists between the total HOMED10 values estimated in water solution and in the gas phase for the neutral and ionized tautomers of the parent system − purine (ten bonds). This relationship is also present in Fig. S2 for comparison. The HOMED10 values for the purine isomers were taken from ref. [25]. A similar tendency was found for correlations between the relative energies (Δ*E*) in the gas phase and in water solution for the neutral adenine and purine tautomers (Fig. S3, Supplementary material). However, for the ionized forms the changes in the relative energies are not parallel to those for the neutral forms and any common linear relation can be proposed.

### HOMED/ΔE relation

Adenine is a nice example of natural product with a good relationship observed between prototropy and electron delocalization for the isolated and hydrated neutral tautomers. The relative energies (Δ*E*), which measure the relative stabilities of individual tautomers, correlate well with the total HOMED11 indices, which measure the geometric consequences of electron delocalization for the whole tautomeric system − 11 bonds (Fig. 1). The relative energies for the hydrated adenine isomers were taken from ref. [15]. The HOMED and Δ*E* values for the isolated (gas phase) adenine isomers were taken from refs. [12, 15]. The good HOMED/Δ*E* relationship confirms that electron delocalization, and particularly aromaticity, is one of the main factors that dictates the tautomeric preferences for neutral adenine in water solution and in the gas phase. Solvent reduces solely the internal effects between the functional groups and diminishes the deviations of points for particular isomers from the HOMED/Δ*E* relation.

Direct comparison of the Δ*E* values calculated for the hydrated tautomers of adenine with the partial HOMED values estimated for their fragments (five, six, seven, and ten bonds) shows additionally that the variations of the HOMED5 indices for the imidazole fragment do not follow well the variations of the Δ*E* values, and a scatter plot is rather observed in water solution (Fig. S4a, Supplementary material). The main reason is a small variation of the position of the labile proton(s) for the imidazole fragment [15]. There are 23 tautomers of the bicyclic adenine possessing different stabilities (Δ*E* values vary from 0 to 68 kcal mol^−1^ in water solution [15]), whereas these tautomers contain solely ten different imidazole fragments, for which the HOMED5 values vary in four ranges in water solution: 0.89-0.96, 0.56-0.67, 0.43-0.49, and 0.33-0.39, respectively. The fragments similar to the free imidazole tautomers possess the HOMED5 values very close to those estimated in water solution for the imidazole N1H/N3H (0.917), C2H (0.354), and C4H/C5H tautomers (0.390) [67]. The small variations of the HOMED5 values for the adenine tautomers affect the deviations of points from the linear HOMED/Δ*E* relation for the purine fragment (Fig. S4d), and also for the whole adenine tautomeric system (Fig. 1). Similar tendency has been found in the gas phase between the HOMED11 and Δ*G* values [12].

The HOMED6 (Fig. S4b) and HOMED7 (Fig. S4c) values estimated for the pyrimidine and 4-aminopyrimidine fragments of the adenine tautomers, seem to be almost parallel to the Δ*E* values in water solution. The variations of the position of the labile proton(s) in these fragments are considerably greater than that for the imidazole one. The 23 adenine tautomers contain 19 different 4-aminopyrimidine fragments. The HOMED7 values progressively change from 0.68 to 0.99 for the 4-aminopyrimidine fragments with the labile proton(s) at the N atom(s) and from 0.33 to 0.73 for the 4-aminopyrimidine fragments containing at least one labile proton at the C atom. Some subtle internal effects of the exo−NH_2_/=NH group cause some deviations of points for particular isomers from the HOMED/Δ*E* relation. These subtle effects also influence the deviations of points from the HOMED/Δ*E* relation for the whole adenine tautomeric system (Fig. 1). This tendency observed in water solution is similar to that in the gas phase [12].

### Effects of the exo functional group

Total effects of the exo−NH_2_ group in water solution are different than those in the gas phase (Table S5, Supplementary material). In the gas phase [12], the total effects are a mixture of the inductive and resonance effects of the − N10H_2_ group and also of those resulting from specific intramolecular interactions of this group with the endo neighboring N1/N1H and N7/N7H groups (Fig. S1, Supplementary material). In water solution, the total effects of the − N10H_2_ group additionally contain specific interactions with solvent. These interactions change the partial N10H_2_ effects possible in the gas phase, and change the total N10H_2_ effects in water solution. To quantitatively measure the total N10H_2_ effects for neutral adenine, the HOMED indices (for the five, six, and ten bonds) and the relative energies (Δ*E*), estimated for the amine tautomers of adenine (**A1**-**A9**) were compared with those for the parent system – purine (**P1**-**P9** - analogous to **A1**-**A9**) [25], both calculated at the same levels of theory, in the gas phase and in water solution. The comparisons lead to the geometric {δHOMED10 = HOMED10(**A**) − HOMED10(**P**)} and energetic {δ*E* = Δ*E*(**A**) − Δ*E*(**P**)} total N10H_2_ effects. Their variations in water solution and in the gas phase are illustrated in Fig. 2.

The electron-withdrawing inductive effect of the N10H_2_ group is energetically unfavorable. In the gas phase, this effect is well manifested for **A6**, for which the n-π conjugation between the N10H_2_ group and the purine system is not possible [15]. Moreover, the N10H_2_ group cannot intramolecularly interact with the endo functional groups [15]. The electron-withdrawing inductive effect of the N10H_2_ group strongly reduces the basicity of the C6 atom containing the N10H_2_ group such that this atom is exceptionally rare and may take the labile proton. Consequently, the δ*E* value is strongly positive in the gas phase (19.1 kcal mol^−1^). It is slightly lower in water solution (17.9 kcal mol^−1^) due to interactions of the N10H_2_ group with the polar solvent. The solvent also diminishes the geometric N10H_2_ effect. The absolute δHOMED10 value decreases when going from the gas phase to water solution (by 0.019 units).

The electron-donating resonance effect of the N10H_2_ group possible for the other amine tautomers of adenine (**A1**-**A5** and **A7**-**A9**) depends on the position of the labile proton [15]. It is less or more energetically favorable. On the other hand, the specific intramolecular interactions of the N10H_2_ group with the N1/N1H and N7/N7H groups seem to be energetically favorable solely for **A2**-**A5**, **A8**, and **A9** (Fig. S1, Supplementary material). For **A1** and **A7**, these interactions are energetically unfavorable due to some repulsions of the N10H/N1H and N10H/N7H groups, respectively. These unfavorable effects lead to the large positive δ*E* values for **A1** (5.6 kcal mol^−1^) and **A7** (4.3 kcal mol^−1^) in the gas phase. In water solution, these effects are reduced by energetically favorable interactions with the polar solvent, and the δ*E* values decrease (to 1.9 and 1.8 kcal mol^−1^, respectively). The energetically unfavorable intramolecular interactions of the N10H and N1H groups in **A1**, and the N10H and N7H groups in **A7** do not affect very much electron delocalization for the pyrimidine and imidazole fragments of **A1** and **A7**, respectively (Table S5, Supplementary material).

Interestingly, the geometric and energetic total effects of the exo−NH_2_ group are strongly favorable for some NH-CH tautomers: **A4**, **A5**, and **A8**. The δHOMED values are very large in the gas phase and also in water solution. The δ*E* values are even more negative in water solution (−7.0, −13.2, and −9.1 kcal mol^−1^) than in the gas phase (−4.5, −10.1, and −6.1 kcal mol^−1^, respectively). The largest favorable geometric and energetic effects take place for **A5**, indicating that the exo−NH_2_ group strongly favors the transfer of the proton to the C5 atom of adenine in comparison to purine. This atom is also favored for the labile proton in uric acid [68]. The particular stability of the C5H tautomer for uric acid may partially explain the formation of the unstable intermediate, 5-hydroxyisourate with the OH group at the C5 atom [15, 69–72].

**Fig. 3:**
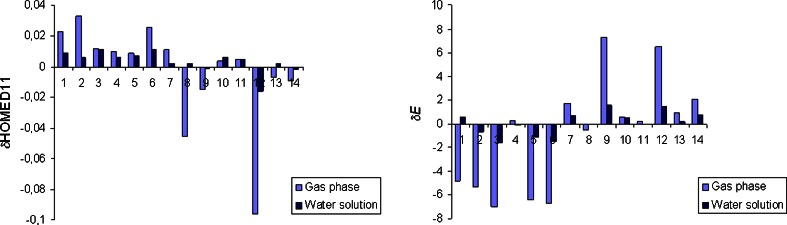
Variations of the geometric (δHOMED11) and energetic (δ*E* in kcal mol^−1^) total effects of the exo = NH group for the neutral imine tautomers of adenine in the gas phase and in water solution. Numbers 1–14 correspond to the imine tautomers **A10**-**A23**, respectively

For the imine tautomers of adenine (**A10**-**A23**, Fig. S1 in Supplementary material), the imine H atom in the exo = NH group may take two different positions (**a** and **b**). This leads to various specific intramolecular interactions between the exo and endo functional groups which affect the energetic and geometric parameters of individual isomers (Table S6, Supplementary material). When intramolecular interactions are energetically favorable for one of the isomers and energetically unfavorable for the other one, e.g., for **A10**, **A11**, **A12**, **A14**, **A15**, **A18**, and **A21**, the difference between the relative energies of these isomers {δ*E* = Δ*E*(**b**) − Δ*E*(**a**)} is exceptionally large in the gas phase (4–7 kcal mol^−1^). Solvent diminishes intramolecular interactions and reduces the δ*E* values (to 1–2 kcal mol^−1^). When there are no significant differences between intramolecular interactions for the isomers **a** and **b**, the δ*E* values are close to zero in water solution and not larger than 2 kcal mol^−1^ in the gas phase. Unfortunately, the variations of the energetic parameters are not in line with those of the geometric ones (Fig. 3). However, the differences between the total HOMED indices estimated for the isomers **a** and **b** {δHOMED11 = HOMED(**b**) – HOMED11(**a**)} are lower in water solution than in the gas phase, and thus the deviations of points for the hydrated imine isomers in the HOMED/Δ*E* plot are smaller than those for the isolated ones (Fig. 1).

## Conclusions

Quantum-chemical calculations, performed for the favored and rare tautomers of neutral and ionized adenine in aqueous solution {PCM(water)//DFT(B3LYP)/6-311+G(d,p)} and compared to those in the gas phase {DFT(B3LYP)/6-311+G(d,p)}, indicate that solvation and ionization effects influence the geometric parameters less drastically than the energetic ones. The geometry-based HOMED indices estimated here for the neutral and ionized forms of adenine in aqueous solution are parallel to those reported previously for the isolated molecules (gas phase) [12]. Ionization effects on the HOMED indices seem to be independent of environment. Quite a different situation takes place for the relative energies. A good relation between the relative energies found in the gas phase and in aqueous solution exists solely for the neutral adenine tautomers. For the ionized forms, no common linear relation can be proposed.

Adenine is a good example of natural product for which a good relation between prototropy and electron delocalization for all possible tautomers of neutral adenine can be proposed. The relative energies, which measure the tautomeric conversions, correlate well with the HOMED indices, which measure the geometric consequences of electron delocalization for individual tautomers. The solvent diminishes the subtle internal effects between the functional groups making the deviations of some isomers from the linear relationship smaller in aqueous solution than those in the gas phase. Aromaticity is the main factor that dictates the tautomeric preferences for neutral adenine similar to its parent system – purine [25]. Predominance of the aromatic NH-NH tautomers in the tautomeric mixture of neutral adenine seems to be independent of environment. Positive ionization (one-electron oxidation) does not change this tendency, and resonance conjugations seem to play the principal role for the oxidized adenine tautomers. Negative ionization (one-electron reduction) increases the stability of the non-aromatic NH-CH tautomers (considered as the rare forms for neutral adenine) that some of them are favored in the tautomeric mixture. In this case, the electron affinity is a more important factor than aromaticity and influences the tautomeric preferences [15]. However, the variations of the energetic parameters for the negatively ionized adenine NH-NH and NH-CH tautomers do not significantly change the order of their electron delocalizations.

## Electronic supplementary material

Below is the link to the electronic supplementary material.

ESM 1 (DOC 461 kb)

## References

[1] Elguero J, Marzin C, Katritzky AR, Linda P (1976) The tautomerism of heterocycles. Academic, New York

[2] Pozharskii AF, Soldatenkov AT, Katritzky AR (1997). Heterocycles in life and society.

[3] Raczyńska ED, Kosińska W, Ośmiałowski B, Gawinecki R (2005). Tautomeric equilibria in relation to pi-electron delocalization. Chem Rev.

[4] Kwiatkowski JS, Person WB, Beveridge DL, Lavery R (1990). Theoretical biochemistry and molecular biology.

[5] Pauling L (1960). The Nature of the Chemical Bonds.

[6] Cyrański MK, Gilski M, Jaskólski M, Krygowski TM (2003). On the aromatic character of the heterocyclic bases of DNA and RNA. J Org Chem.

[7] Cysewski P (2005). An ab initio study on nucleic acid bases aromaticities. J Mol Struct (Theochem).

[8] Cysewski P, Szefler B (2010). Environment influences on the aromatic character of nucleobases and amino acids. J Mol Model.

[9] Huertas O, Poater J, Fuentes-Cabrera M, Orozco M, Solà M, Luque FJ (2006). Local aromaticity in natural nucleobases and their size-expanded benzo-fused derivatives. J Phys Chem A.

[10] Vogt N, Dorofeeva OV, Sipachev VA, Rykov NA (2009). Molecular structure of 9H-adenine tautomer: gas-phase electron diffraction and quantum-chemical studies. J Phys Chem A.

[11] Malikova K, Novosadova L, Pipiska M, Marek R (2011). Chemical shift tensors in isomers of adenine: relation to aromaticity of purine rings. Chem Phys Chem.

[12] Raczyńska ED, Kolczyńska K, Stępniewski TM, Kamińska B (2013). On relation between prototropy and electron delocalization for neutral and redox adenine – DFT studies. Comput Theor Chem.

[13] Raczyńska ED, Krygowski TM, Duczmal K, Hallmann M (2006) On geometry-based HOMED (a measure of hyperconjugation, n-π, and π-π conjugation) and HOMA index (a measure of aromaticity). XVIII International Conference on Physical Organic Chemistry, Warsaw, p. 31 (Book of abstracts)

[14] Raczyńska ED, Hallmann M, Kolczyńska K, Stępniewski TM (2010). On the harmonic oscillator model of electron delocalization (HOMED) index and its application to heteroatomic π-electron systems. Symmetry.

[15] Raczyńska ED, Makowski M, Zientara-Rytter K, Kolczyńska K, Stępniewski TM, Hallmann M (2013). Quantum-chemical studies on the favored and rare tautomers of neutral and redox adenine. J Phys Chem A.

[16] Kruszewski J, Krygowski TM (1972). Definition of aromaticity basing on the harmonic oscillator model. Tetrahedron Lett.

[17] Krygowski TM, Kruszewski J (1974). Aromaticity of thiophene, pyrrole and furan in terms of aromaticity indices and Hammett ρ constants. Bull Acad Pol Sci Sér Sci Chim.

[18] Krygowski TM, Cyrański MK (2001). Structural aspects of aromaticity. Chem Rev.

[19] Cyranski MK (2005). Energetic aspects of cyclic π-electron delocalization: evaluation of the methods of estimating aromatic stabilization energies. Chem Rev.

[20] Katritzky AR, Jug K, Oniciu DC (2001). Quantitative measures of aromaticity for mono-, bi-, and tricyclic penta- and hexaatomic heteroaromatic ring systems and their interrelationships. Chem Rev.

[21] Chen Z, Wannere CS, Corminboeuf C, Puchta R, Schleyer PR (2005). Nucleus-independent chemical shifts (NICS) as an aromatic criterion. Chem Rev.

[22] Poater J, Dusan M, Solà M, Silvi B (2005). Theoretical evaluation of electron delocalization in aromatic molecules by means of atoms in molecules (AIM) and electron localization function (ELF) topological approaches. Chem Rev.

[23] Krygowski TM (1993). Crystallographic studies of inter- and intramolecular interactions reflected in aromatic character of π-electron systems. J Chem Inf Comput Sci.

[24] Frizzo CP, Martins MP (2012). Aromaticity in heterocycles: new HOMA index parametrization. Struct Chem.

[25] Raczyńska ED, Kamińska B (2013). Variations of the tautomeric preferences and π-electron delocalization for the neutral and redox forms of purine when proceeding from the gas phase (DFT) to water (PCM). J Mol Model.

[26] Krygowski TM, Oziminski WP, Cyrański MK (2012). Aromatic character of heptafulvene and its complexes with halogen atoms. J Mol Model.

[27] Parr RG, Yang W (1989). Density functional theory of atoms and molecular orbital theory.

[28] Becke AD (1993). Density-functional thermochemistry. III. The role of exact exchange. J Chem Phys.

[29] Lee C, Yang W, Parr RG (1988). Development of the colle-salvetti correlation-energy formula into a functional of the electron density. Phys Rev B.

[30] Hehre WJ, Radom L, Schleyer PR, Pople JA (1986). Ab initio molecular theory.

[31] Miertus S, Tomasi J (1982). Approximate evaluation of the electrostatic free energy and internal energy changes in solution processes. Chem Phys.

[32] Miertus S, Scrocco E, Tomasi J (1981). Electrostatic interaction of a solute with a continuum. Adirect utilization of ab initio molecular potentials for the prevision of solvent effects. Chem Phys.

[33] Frisch MJ, Trucks GW, Schlegel HB, Scuseria GE, Robb MA, Cheeseman JR, Montgomery JA, Vreven T, Kudin KN, Burant JC, Millam JM, Iyengar SS, Tomasi J, Barone V, Mennucci B, Cossi M, Scalmani G, Rega N, Petersson R, Nakatsuji H, Hada M, Ehara M, Toyota K, Fukuda R, Hasegawa J, Ishida M, Nakajima T, Honda Y, Kitao O, Nakai H, Klene M, Li X, Knox JE, Hratchian HP, Cross JB, Bakken V, Adamo C, Jaramillo R, Gomperts R, Stratmann RE, Yazyev O, Austin AJ, Cammi R, Pomelli C, Ochterski JW, Ayala PY, Morokuma K, Voth GA, Salvador P, Dannenberg JJ, Zakrzewski VG, Dapprich S, Daniels AD, Strain MC, Farkas O, Malick DK, Rabuck AD, Raghavachari K, Foresman JB, Oritz JV, Cui Q, Baboul AG, Clifford S, Cioslowski J, Stefanov BB, Liu G, Liashenko A, Piskorz P, Komaromi I, Martin RL, Fox DJ, Keith T, Al-Laham MA, Peng CY, Nanayakkara A, Challacombe M, Gill PMW, Johnson B, Chen W, Wong MW, Gonzalez C, Pople JA (2004). Gaussian-03, Revision E.01.

[34] Katritzky AR, Karelson M (1991). AM1 calculations of reaction field effects on the tautomeric equilibria of nucleic acid pyrimidine and purine bases and their 1-methyl analogs. J Am Chem Soc.

[35] Ha T-K, Keller MJ, Gunde R, Gunthard HH (1996). Quantum chemical study of structure, energy, rotational constants, electric dipole moments and electric field gradients of all isomeric adenines. J Mol Struct (Theochem).

[36] Hanus M, Kabeláč M, Rejnek J, Ryjáček F, Hobza P (2004). Calculated ab initio study of nucleic acid bases and their tautomers in the gas phase, in a microhydrated environment, and in aqueous solution. Part 3. Adenine. J Phys Chem B.

[37] Guerra CF, Bickelhaupt FM, Saha S, Wang F (2006). Adenine tautomers: relative stabilities, ionization energies, and mismatch with cytosine. J Phys Chem A.

[38] Singh RK, Oritz JV, Mishra MK (2010). Tautomeric forms of adenine: vertical ionization energies and Dyson orbitals. Int J Quantum Chem.

[39] Laxer A, Major DT, Gottlieb HE, Fischer B (2001). (^15^ N_5_)-Labeled adenine derivatives: synthesis and studies of tautomerism by ^15^ N NMR spectroscopy and theoretical calculations. J Org Chem.

[40] Haranczyk M, Gutowski M, Li X, Bowen KH (2007). Bound anionic states of adenine. Proc Natl Acad Sci U S A.

[41] Li X, Bowen KH, Haranczyk M, Bachorz RA, Mazurkiewicz K, Rak J, Gutowski M (2007) Photoelectron spectroscopy of adiabadically bound valence anions of rare tautomers of the nucleic adic bases. J Chem Phys 127:174309/1-6 10.1063/1.2795719 17994820

[42] Watson JD, Crick FHC (1953). Genetical implications of the structure of deoxyribonucleic acid. Nature.

[43] Sun G, Nicklaus MC (2007). Natural structures and aromaticity of the nucleobases. Theor Chem Acc.

[44] Brown RD, Godfrey PD, McNaughton DD, Pierlot AA (1989). A study of the major gas-phase tautomer of adenine by microwave spectroscopy. Chem Phys Lett.

[45] Majoube M, Millié P, Lagant P, Vergoten G (1994). Resonance Raman enhancement for adenine and guanine residues. J Raman Spectrosc.

[46] Schoone K, Houben L, Smets J, Adamowicz L, Maes G (1996). Matrix-isolation FT-IR and ab initio 6-31++G** study of 1-CH_3_-adenine tautomerism. Spectrochim Acta A.

[47] Colarusso P, Zhang K, Guo B, Bernath PF (1997). The infrared spectra of uracil, thymine and adenine in the gas phase. Chem Phys Lett.

[48] Broo A (1998). A theoretical investigation of the physical reason for the very different luminescence properties of two isomers adenine and 2-aminopurine. J Phys Chem A.

[49] Kim NJ, Jeong G, Kim YS, Sung J, Kim SK, Park YD (2000). Resonant two-photon ionization and laser induced fluorescence spectroscopy of jet-cooled adenine. J Chem Phys.

[50] Mishra SK, Shukla MK, Mishra PC (2000). Electronic spectra of adenine and 2-aminopurine: an ab initio study of energy level diagrams of different tautomers in the gas phase and aqueous solution. Spectrochim Acta A.

[51] Mennucci B, Toniolo A, Tomasi J (2001). Theoretical study of the photophysics of adenine in solution: tautomerism, deactivation mechanisms, and comparison with the 2-aminopurine fluorescent isomer. J Phys Chem A.

[52] Plutzer C, Nir E, de Vries MS, Kleinermanns K (2001). IR-UV double-resonance spectroscopy of the nucleobase adenine. Phys Chem Chem Phys.

[53] Plutzer C, Kleinermanns K (2002). Tautomers and electronic states of jet-cooled adenine investigated by double resonance spectroscopy. Phys Chem Chem Phys.

[54] Wang F, Downton MT, Kidwani N (2005) Adenine tautomer electronic structural signatures studies using dual space analysis. J Theor Comput Chem 4:247–264

[55] Nowak MJ, Lapinski L, Kwiatkowski JS (1989). An infrared matrix isolation study of tautomerism in purine and adenine. Chem Phys Lett.

[56] Czermiński R, Szczepaniak K, Person WB, Kwiatkowski JS (1990). Intermolecular interactions and tautomerism of nucleic acid bases and their analogues. J Mol Struct.

[57] Nowak MJ, Lapinski L, Kwiatkowski JS, Leszczynski J (1991). Infrared matrix isolation and ab initio quantum mechanical studies of purine and adenine. Spectrochim Acta A.

[58] Nowak MJ, Rostkowska H, Lapinski L, Kwiatkowski JS, Leszczynski J (1994). Experimental matrix isolation and theoretical ab initio HF/6–31G(d, p) studies of infrared spectra of purine, adenine and 2-chloroadenine. Spectrochim Acta A.

[59] Nowak MJ, Rostkowska H, Lapinski L, Kwiatkowski JS, Leszczynski J (1994). Tautomerism N(9)H → N(7)H of purine, adenine, and 2-chloroadenine. Combined experimental IR matrix isolation and ab initio quantum mechanical studies. J Phys Chem.

[60] Nowak MJ, Lapinski L, Kwiatkowski JS, Leszczynski J (1996). Molecular structure and infrared spectra of adenine. Experimental-matrix isolation and density functional theory study of adenine N-15 isotopomers. J Phys Chem A.

[61] Gu JD, Leszczynski J (1999). A DFT study of the water-assisted intramolecular proton transfer in the tautomers of adenine. J Phys Chem A.

[62] Sukhanov OS, Shishkin OV, Grob L, Podolyan Y, Leszczynski J (2003). Molecular structure and hydrogen bonding in polyhydrated complexes of adenine: a DFT study. J Phys Chem B.

[63] Close DM, Crespo-Hernandez CE, Grob L, Leszczynski J (2008). Ionization energy thresholds of microhydrated adenine and its tautomers. J Phys Chem A.

[64] Periquet V, Moreau A, Carles S, Schermann JP, Desfrancois C (2000). Cluster size effects upon anion solvation of N-heterocyclic molecules and nucleic acid bases. J Electron Spectrosc Relat Phenom.

[65] Jalbout AF, Adamowicz L (2001). Dipole-bound anions of adenine-water clusters. Ab initio study. J Phys Chem A.

[66] Nugent ML, Adamowicz L (2005). Stabilization of the adenine covalent anion by micro-hydration: theoretical study. Mol Phys.

[67] Raczyńska ED (2012). Quantum-chemical studies of the consequences of one-electron oxidation and one-electron reduction for imidazole in the gas phase and in water. Comput Theor Chem.

[68] Raczyńska ED, Makowski M, Szeląg M, Kamińska B, Zientara K (2010). Importance of CH tautomers in the tautomeric mixture of uric acid. J Mol Struct (Theochem).

[69] Ramazzina I, Folli C, Secchi A, Berni R, Percudani R (2006). Completing the uric acid degradation pathway through phylogenetic comparison of whole genomem. Nat Chem Biol.

[70] Colloc’h N, El Hajji M, Bachet B, L’Hermete G, Schiltz M, Prangé T, Castro B, Mornon J-P (1997). Crystal structure of the protein drug urate oxidase-inhibitor complex at 2.05 Å resolution. Nat Struct Biol.

[71] Retailleau P, Colloc’h N, Vivares D, Bonneté F, Castro B, El Hajji M, Mornon J-P, Monard G, Prangé T (2004). Complexed and ligand-free high resolution structures of urate oxidase (Uox) from Aspergillus flavus: a reassignment of the active-site binding. Acta Cryst D.

[72] Retailleu P, Colloc’h N, Vivares D, Bonneté F, Castro B, El Hajji M, Prangé T (2004). Urate oxidase from Aspergillus flavus: new crystalpacking contacts in relation to the content of the active site. Acta Cryst D.

