# Supplementary material for: Geometric consequences of electron delocalization for adenine tautomers in aqueous solution
Source: J Mol Model. 2014 May 15;20(6):2234. doi: 10.1007/s00894-014-2234-4 (PMC4072068; doi:10.1007/s00894-014-2234-4)
Supplement: Supplementary file 1 — (DOC 461 kb) [file 894_2014_2234_MOESM1_ESM.doc]

Supplementary Material

Geometric consequences of electron delocalization
for adenine tautomers in water solution

Ewa D. Raczyńska,1* Mariusz Makowski2

*1Department of Chemistry,Warsaw University of Life Sciences (SGGW),
ul. Nowoursynowska 159c, 02-776 Warszawa, Poland*

*2Laboratory of Intermolecular Interactions, Faculty of Chemistry, Univeristy of Gdańsk,
ul. W. Stwosza 63, 80-308 Gdańsk, Poland*

The CC and CN bond lengths calculated at the PCM(water)//DFT(B3LYP)/6-311+G(d,p) level for the neutral isomers of adenine (Fig. S1) are given in Table S1. Generally, the CC and CN bond lengths vary from 1.38 to 1.52 Å and from 1.27 to 1.47 Å, respectively. For the NH-NH tautomers, the CC (1.38-1.44 Å) and CN (1.29-1.39 Å) bond lengths are not very different from those calculated at the same level of theory for fully delocalized benzene (1.40 Å) and 1,3,5-triazine (1.34 Å), confirming their aromatic character. For the NH-CH tautomers, the CC bond lengths (1.45-1.52 Å) are close to that for ethane (1.53 Å), and the CN bond lengths (1.35-1.47 Å and 1.27-1.33 Å) are close to those for methylamine (1.46 Å) and methylimine (1.27 Å), indicating weak π-electron delocalization. Positive and negative ionizations affect the CC and CN bond lengths (Table S2). For the selected radical cations, the CC bond lengths vary from 1.42 to 1.46 Å, whereas those of CN from 1.29 to 1.41 Å. For the selected radical anions, the variations are as follows: 1.39-1.51 and 1.30-1.48 Å, respectively. All changes of the CC and CN bond lengths when going from the neutral to ionized forms of adenine indicate great sensitivity of π-electron delocalization on electron- and proton-transfer. To quantitatively measure of these changes, the geometry-based HOMED index was applied to the bond lengths calculated in water solution and compared to those found in the gas phase (Tables S3 and S4). Intramolecular effects were also compared for the neutral isomers (Tables S5 and S6). These effects influence the relation between the HOMED indices (Fig. S2) as well as between the relative energies in both environments (Fig. S3). They also affect the relation between the HOMED indices and the relative energies (Fig. S4).

**Figure S1**  The amine and imine isomers of adenine and possible favorable intramolecular interactions

(a) amine tautomers

|  |  |  |
| --- | --- | --- |
| **A1** | **A2** | **A3** |
|  |  |  |
| **A4** | **A5** | **A6** |
|  |  |  |
| **A7** | **A8** | **A9** |

(b) imine tautomers

|  |  |  |  |
| --- | --- | --- | --- |
| **A10a** | **A10b** | **A11a** | **A11b** |
|  |  |  |  |
| **A12a** | **A12b** | **A13a** | **A13b** |
|  |  |  |  |
| **A14a** | **A14b** | **A15a** | **A15b** |
|  |  |  |  |
| **A16a** | **A16b** | **A17a** | **A17b** |
|  |  |  |  |
| **A18a** | **A18b** | **A19a** | **A19b** |
|  |  |  |  |
| **A20a** | **A20b** | **A21a** | **A21b** |
|  |  |  |  |
| **A22a** | **A22b** | **A23a** | **A23b** |

**Table S1**  The CC and CN bond lengths for the neutral adenine isomers in water solution

| Isomer | N1C2 | C2N3 | N3C4 | C4C5 | C5C6 | C6N1 | C5N7 | N7C8 | C8N9 | N9C4 | C6N10 |
| --- | --- | --- | --- | --- | --- | --- | --- | --- | --- | --- | --- |
| **A1** | 1.37 | 1.30 | 1.37 | 1.42 | 1.40 | 1.37 | 1.37 | 1.34 | 1.36 | 1.35 | 1.34 |
| **A2** | 1.43 | 1.45 | 1.28 | 1.47 | 1.45 | 1.30 | 1.30 | 1.42 | 1.30 | 1.40 | 1.35 |
| **A3** | 1.31 | 1.35 | 1.37 | 1.41 | 1.40 | 1.37 | 1.38 | 1.33 | 1.37 | 1.34 | 1.34 |
| **A4** | 1.39 | 1.29 | 1.45 | 1.49 | 1.47 | 1.32 | 1.29 | 1.43 | 1.28 | 1.46 | 1.33 |
| **A5** | 1.36 | 1.32 | 1.36 | 1.50 | 1.50 | 1.33 | 1.46 | 1.30 | 1.41 | 1.31 | 1.32 |
| **A6** | 1.28 | 1.42 | 1.28 | 1.49 | 1.48 | 1.46 | 1.29 | 1.43 | 1.30 | 1.40 | 1.45 |
| **A7** | 1.34 | 1.33 | 1.35 | 1.40 | 1.41 | 1.35 | 1.38 | 1.36 | 1.32 | 1.38 | 1.35 |
| **A8** | 1.37 | 1.31 | 1.37 | 1.47 | 1.47 | 1.32 | 1.28 | 1.47 | 1.45 | 1.30 | 1.32 |
| **A9** | 1.34 | 1.33 | 1.34 | 1.40 | 1.41 | 1.34 | 1.38 | 1.31 | 1.38 | 1.37 | 1.35 |
| **A10a** | 1.46 | 1.43 | 1.27 | 1.48 | 1.47 | 1.37 | 1.29 | 1.43 | 1.29 | 1.41 | 1.28 |
| **A10b** | 1.46 | 1.43 | 1.27 | 1.48 | 1.47 | 1.37 | 1.29 | 1.43 | 1.29 | 1.41 | 1.28 |
| **A11a** | 1.38 | 1.28 | 1.45 | 1.50 | 1.48 | 1.40 | 1.29 | 1.43 | 1.28 | 1.46 | 1.27 |
| **A11b** | 1.38 | 1.28 | 1.45 | 1.50 | 1.48 | 1.40 | 1.29 | 1.43 | 1.28 | 1.46 | 1.27 |
| **A12a** | 1.36 | 1.31 | 1.36 | 1.51 | 1.51 | 1.41 | 1.45 | 1.29 | 1.41 | 1.30 | 1.26 |
| **A12b** | 1.36 | 1.31 | 1.36 | 1.51 | 1.51 | 1.41 | 1.45 | 1.29 | 1.41 | 1.30 | 1.27 |
| **A13a** | 1.37 | 1.30 | 1.38 | 1.39 | 1.43 | 1.41 | 1.37 | 1.36 | 1.32 | 1.37 | 1.29 |
| **A13b** | 1.36 | 1.30 | 1.37 | 1.39 | 1.44 | 1.41 | 1.37 | 1.36 | 1.32 | 1.37 | 1.29 |
| **A14a** | 1.37 | 1.30 | 1.38 | 1.48 | 1.48 | 1.40 | 1.28 | 1.46 | 1.45 | 1.29 | 1.27 |
| **A14b** | 1.36 | 1.30 | 1.38 | 1.48 | 1.48 | 1.40 | 1.28 | 1.46 | 1.45 | 1.29 | 1.27 |
| **A15a** | 1.36 | 1.30 | 1.36 | 1.39 | 1.44 | 1.42 | 1.38 | 1.32 | 1.37 | 1.37 | 1.29 |
| **A15b** | 1.36 | 1.30 | 1.36 | 1.39 | 1.44 | 1.41 | 1.38 | 1.32 | 1.37 | 1.37 | 1.29 |
| **A16a** | 1.31 | 1.35 | 1.45 | 1.50 | 1.48 | 1.40 | 1.29 | 1.43 | 1.28 | 1.46 | 1.28 |
| **A16b** | 1.30 | 1.35 | 1.44 | 1.50 | 1.48 | 1.40 | 1.29 | 1.43 | 1.28 | 1.46 | 1.28 |
| **A17a** | 1.29 | 1.38 | 1.36 | 1.50 | 1.51 | 1.42 | 1.46 | 1.29 | 1.42 | 1.30 | 1.27 |
| **A17b** | 1.29 | 1.38 | 1.35 | 1.50 | 1.52 | 1.41 | 1.46 | 1.29 | 1.42 | 1.30 | 1.27 |
| **A18a** | 1.30 | 1.36 | 1.37 | 1.38 | 1.44 | 1.41 | 1.38 | 1.35 | 1.33 | 1.36 | 1.29 |
| **A18b** | 1.30 | 1.36 | 1.37 | 1.38 | 1.44 | 1.41 | 1.38 | 1.35 | 1.33 | 1.36 | 1.29 |
| **A19a** | 1.30 | 1.37 | 1.37 | 1.47 | 1.48 | 1.41 | 1.28 | 1.46 | 1.46 | 1.28 | 1.28 |
| **A19b** | 1.29 | 1.37 | 1.37 | 1.47 | 1.49 | 1.40 | 1.28 | 1.46 | 1.46 | 1.28 | 1.28 |
| **A20a** | 1.30 | 1.36 | 1.37 | 1.38 | 1.45 | 1.42 | 1.38 | 1.31 | 1.38 | 1.36 | 1.29 |
| **A20b** | 1.30 | 1.36 | 1.36 | 1.38 | 1.45 | 1.42 | 1.38 | 1.31 | 1.38 | 1.36 | 1.29 |
| **A21a** | 1.30 | 1.38 | 1.30 | 1.51 | 1.51 | 1.40 | 1.45 | 1.33 | 1.34 | 1.36 | 1.27 |
| **A21b** | 1.30 | 1.38 | 1.30 | 1.51 | 1.52 | 1.40 | 1.45 | 1.33 | 1.33 | 1.36 | 1.27 |
| **A22a** | 1.30 | 1.39 | 1.30 | 1.50 | 1.51 | 1.41 | 1.46 | 1.28 | 1.40 | 1.35 | 1.27 |
| **A22b** | 1.30 | 1.39 | 1.30 | 1.50 | 1.52 | 1.41 | 1.46 | 1.28 | 1.40 | 1.35 | 1.27 |
| **A23a** | 1.31 | 1.37 | 1.31 | 1.48 | 1.48 | 1.40 | 1.28 | 1.45 | 1.46 | 1.33 | 1.28 |
| **A23b** | 1.31 | 1.38 | 1.31 | 1.48 | 1.48 | 1.40 | 1.28 | 1.45 | 1.46 | 1.33 | 1.28 |

**Table S2**  The CC and CN bond lengths for the selected radical cations and radical anions of adenine in water solution

| Isomer | N1C2 | C2N3 | N3C4 | C4C5 | C5C6 | C6N1 | C5N7 | N7C8 | C8N9 | N9C4 | C6N10 |
| --- | --- | --- | --- | --- | --- | --- | --- | --- | --- | --- | --- |
| **A1+·** | 1.36 | 1.32 | 1.33 | 1.46 | 1.43 | 1.37 | 1.31 | 1.41 | 1.32 | 1.36 | 1.31 |
| **A3+·** | 1.30 | 1.37 | 1.34 | 1.45 | 1.44 | 1.37 | 1.32 | 1.39 | 1.34 | 1.34 | 1.31 |
| **A7+·** | 1.31 | 1.37 | 1.31 | 1.44 | 1.43 | 1.36 | 1.34 | 1.40 | 1.30 | 1.38 | 1.32 |
| **A9+·** | 1.31 | 1.38 | 1.30 | 1.43 | 1.43 | 1.36 | 1.34 | 1.35 | 1.35 | 1.37 | 1.32 |
| **A13a+·** | 1.34 | 1.33 | 1.33 | 1.44 | 1.42 | 1.40 | 1.34 | 1.38 | 1.32 | 1.37 | 1.30 |
| **A13b+·** | 1.33 | 1.33 | 1.33 | 1.44 | 1.42 | 1.40 | 1.34 | 1.38 | 1.31 | 1.37 | 1.30 |
| **A15a+·** | 1.34 | 1.33 | 1.32 | 1.44 | 1.43 | 1.40 | 1.35 | 1.33 | 1.36 | 1.36 | 1.29 |
| **A15b+·** | 1.33 | 1.34 | 1.32 | 1.44 | 1.43 | 1.40 | 1.35 | 1.33 | 1.36 | 1.36 | 1.30 |
| **A18a+·** | 1.30 | 1.37 | 1.34 | 1.42 | 1.42 | 1.39 | 1.35 | 1.37 | 1.32 | 1.35 | 1.31 |
| **A18b+·** | 1.30 | 1.37 | 1.34 | 1.42 | 1.43 | 1.39 | 1.36 | 1.37 | 1.32 | 1.35 | 1.32 |
| **A20a+·** | 1.29 | 1.38 | 1.33 | 1.43 | 1.43 | 1.39 | 1.36 | 1.32 | 1.38 | 1.34 | 1.31 |
| **A20b+·** | 1.29 | 1.38 | 1.33 | 1.42 | 1.43 | 1.39 | 1.36 | 1.32 | 1.38 | 1.35 | 1.31 |
| **A1-·** | 1.36 | 1.31 | 1.39 | 1.41 | 1.43 | 1.42 | 1.37 | 1.36 | 1.35 | 1.36 | 1.42 |
| **A2-·** | 1.46 | 1.46 | 1.32 | 1.43 | 1.44 | 1.30 | 1.36 | 1.36 | 1.34 | 1.38 | 1.38 |
| **A3-·** | 1.33 | 1.42 | 1.40 | 1.39 | 1.44 | 1.34 | 1.38 | 1.35 | 1.37 | 1.35 | 1.40 |
| **A4-·** | 1.37 | 1.31 | 1.46 | 1.50 | 1.40 | 1.35 | 1.35 | 1.38 | 1.31 | 1.47 | 1.37 |
| **A5-·** | 1.37 | 1.36 | 1.33 | 1.51 | 1.50 | 1.32 | 1.47 | 1.32 | 1.37 | 1.36 | 1.37 |
| **A6-·** | 1.30 | 1.38 | 1.34 | 1.43 | 1.50 | 1.47 | 1.34 | 1.38 | 1.34 | 1.38 | 1.48 |
| **A7-·** | 1.33 | 1.35 | 1.38 | 1.42 | 1.39 | 1.39 | 1.38 | 1.40 | 1.35 | 1.35 | 1.42 |
| **A8-·** | 1.34 | 1.33 | 1.37 | 1.45 | 1.42 | 1.35 | 1.33 | 1.47 | 1.47 | 1.32 | 1.37 |
| **A9-·** | 1.33 | 1.38 | 1.35 | 1.40 | 1.43 | 1.35 | 1.37 | 1.34 | 1.39 | 1.37 | 1.42 |
| **A10a-·** | 1.47 | 1.45 | 1.32 | 1.44 | 1.46 | 1.39 | 1.35 | 1.36 | 1.34 | 1.38 | 1.30 |
| **A10b-·** | 1.47 | 1.45 | 1.32 | 1.43 | 1.45 | 1.38 | 1.35 | 1.36 | 1.34 | 1.38 | 1.30 |
| **A14a-·** | 1.36 | 1.31 | 1.38 | 1.45 | 1.44 | 1.41 | 1.33 | 1.47 | 1.47 | 1.31 | 1.31 |
| **A14b-·** | 1.36 | 1.31 | 1.38 | 1.45 | 1.43 | 1.41 | 1.33 | 1.47 | 1.47 | 1.32 | 1.31 |
| **A17a-·** | 1.33 | 1.38 | 1.35 | 1.50 | 1.54 | 1.37 | 1.47 | 1.31 | 1.38 | 1.33 | 1.30 |
| **A19a-·** | 1.30 | 1.37 | 1.38 | 1.44 | 1.44 | 1.41 | 1.34 | 1.47 | 1.48 | 1.30 | 1.32 |
| **A19b-·** | 1.30 | 1.37 | 1.38 | 1.44 | 1.44 | 1.41 | 1.34 | 1.47 | 1.48 | 1.30 | 1.32 |
| **A23a-·** | 1.32 | 1.36 | 1.33 | 1.42 | 1.45 | 1.40 | 1.33 | 1.46 | 1.47 | 1.36 | 1.31 |
| **A23b-·** | 1.32 | 1.36 | 1.33 | 1.42 | 1.45 | 1.39 | 1.34 | 1.46 | 1.47 | 1.36 | 1.31 |

**Table S3**  The partial and total HOMED values and the relative energies (Δ*E* in kcal mol^-1^)a for the neutral adenine isomers in water solution

| Isomer | HOMED5 | HOMED6 | HOMED7 | HOMED10 | HOMED11 | Δ*E* |
| --- | --- | --- | --- | --- | --- | --- |
| **A1** | 0.961 | 0.919 | 0.936 | 0.933 | 0.942 | 6.3 |
| **A2** | 0.668 | 0.466 | 0.576 | 0.542 | 0.603 | 48.8 |
| **A3** | 0.955 | 0.954 | 0.964 | 0.948 | 0.955 | 4.4 |
| **A4** | 0.372 | 0.537 | 0.635 | 0.442 | 0.520 | 50.1 |
| **A5** | 0.484 | 0.668 | 0.734 | 0.603 | 0.658 | 36.4 |
| **A6** | 0.604 | 0.351 | 0.329 | 0.461 | 0.431 | 68.4 |
| **A7** | 0.926 | 0.991 | 0.990 | 0.990 | 0.990 | 2.0 |
| **A8** | 0.378 | 0.797 | 0.839 | 0.578 | 0.636 | 35.4 |
| **A9** | 0.889 | 0.996 | 0.994 | 0.936 | 0.942 | 0.0 |
| **A10a** | 0.589 | 0.350 | 0.459 | 0.444 | 0.502 | 54.4 |
| **A10b** | 0.591 | 0.430 | 0.476 | 0.454 | 0.511 | 55.0 |
| **A11a** | 0.340 | 0.440 | 0.485 | 0.362 | 0.422 | 49.9 |
| **A11b** | 0.337 | 0.420 | 0.499 | 0.369 | 0.428 | 49.2 |
| **A12a** | 0.440 | 0.505 | 0.553 | 0.499 | 0.531 | 38.1 |
| **A12b** | 0.453 | 0.519 | 0.564 | 0.512 | 0.542 | 36.5 |
| **A13a** | 0.944 | 0.837 | 0.845 | 0.884 | 0.872 | 9.1 |
| **A13b** | 0.942 | 0.849 | 0.860 | 0.877 | 0.878 | 9.0 |
| **A14a** | 0.336 | 0.658 | 0.684 | 0.485 | 0.526 | 36.5 |
| **A14b** | 0.333 | 0.677 | 0.699 | 0.493 | 0.533 | 35.4 |
| **A15a** | 0.929 | 0.828 | 0.837 | 0.857 | 0.859 | 9.5 |
| **A15b** | 0.930 | 0.848 | 0.853 | 0.870 | 0.870 | 8.0 |
| **A16a** | 0.340 | 0.458 | 0.534 | 0.405 | 0.463 | 51.2 |
| **A16b** | 0.339 | 0.464 | 0.543 | 0.406 | 0.445 | 51.9 |
| **A17a** | 0.441 | 0.475 | 0.535 | 0.462 | 0.504 | 39.6 |
| **A17b** | 0.457 | 0.470 | 0.532 | 0.464 | 0.506 | 39.6 |
| **A18a** | 0.948 | 0.832 | 0.846 | 0.873 | 0.876 | 12.6 |
| **A18b** | 0.944 | 0.830 | 0.847 | 0.869 | 0.875 | 14.6 |
| **A19a** | 0.325 | 0.661 | 0.693 | 0.468 | 0.516 | 37.9 |
| **A19b** | 0.325 | 0.673 | 0.704 | 0.473 | 0.522 | 38.4 |
| **A20a** | 0.913 | 0.803 | 0.822 | 0.836 | 0.844 | 16.6 |
| **A20b** | 0.914 | 0.811 | 0.830 | 0.841 | 0.849 | 16.6 |
| **A21a** | 0.575 | 0.478 | 0.541 | 0.568 | 0.589 | 39.4 |
| **A21b** | 0.561 | 0.440 | 0.512 | 0.540 | 0.573 | 40.9 |
| **A22a** | 0.432 | 0.462 | 0.528 | 0.462 | 0.505 | 41.0 |
| **A22b** | 0.448 | 0.456 | 0.524 | 0.463 | 0.507 | 41.2 |
| **A23a** | 0.388 | 0.684 | 0.715 | 0.527 | 0.569 | 41.4 |
| **A23b** | 0.388 | 0.682 | 0.716 | 0.523 | 0.567 | 42.2 |

a Data taken from ref. [15]

**Table S4**  Variations of the total HOMED indices for the selected neutral and ionized isomers of adenine when proceeding from the gas phasea to water solution

|  | HOMED11 | |  |  | HOMED11 | |  |
| --- | --- | --- | --- | --- | --- | --- | --- |
| Isomer | Gas | Water | Δb | Isomer | Gas | Water | Δb |
| **A1** | 0.906 | 0.942 | 0.036 | **A15a** | 0.800 | 0.859 | 0.059 |
| **A2** | 0.560 | 0.603 | 0.043 | **A15b** | 0.826 | 0.870 | 0.044 |
| **A3** | 0.944 | 0.955 | 0.011 | **A16a** | 0.403 | 0.463 | 0.060 |
| **A4** | 0.483 | 0.520 | 0.037 | **A16b** | 0.414 | 0.465 | 0.051 |
| **A5** | 0.606 | 0.658 | 0.052 | **A17a** | 0.415 | 0.504 | 0.089 |
| **A6** | 0.364 | 0.431 | 0.067 | **A17b** | 0.370 | 0.506 | 0.136 |
| **A7** | 0.922 | 0.990 | 0.068 | **A18a** | 0.840 | 0.876 | 0.036 |
| **A8** | 0.606 | 0.636 | 0.030 | **A18b** | 0.825 | 0.875 | 0.050 |
| **A9** | 0.938 | 0.942 | 0.004 | **A19a** | 0.453 | 0.516 | 0.063 |
| **A10a** | 0.425 | 0.502 | 0.077 | **A19b** | 0.457 | 0.522 | 0.065 |
| **A10b** | 0.448 | 0.511 | 0.063 | **A20a** | 0.747 | 0.844 | 0.097 |
| **A11a** | 0.345 | 0.422 | 0.077 | **A20b** | 0.752 | 0.849 | 0.097 |
| **A11b** | 0.378 | 0.428 | 0.050 | **A21a** | 0.554 | 0.589 | 0.035 |
| **A12a** | 0.475 | 0.531 | 0.056 | **A21b** | 0.458 | 0.573 | 0.115 |
| **A12b** | 0.487 | 0.542 | 0.055 | **A22a** | 0.413 | 0.505 | 0.092 |
| **A13a** | 0.837 | 0.872 | 0.035 | **A22b** | 0.406 | 0.507 | 0.101 |
| **A13b** | 0.847 | 0.878 | 0.031 | **A23a** | 0.507 | 0.569 | 0.062 |
| **A14a** | 0.479 | 0.526 | 0.047 | **A23b** | 0.498 | 0.567 | 0.069 |
| **A14b** | 0.488 | 0.533 | 0.045 |  |  |  |  |
| **A1+•** | 0.841 | 0.891 | 0.050 | **A15a+•** | 0.870 | 0.909 | 0.039 |
| **A3+•** | 0.878 | 0.900 | 0.022 | **A15b+•** | 0.896 | 0.916 | 0.020 |
| **A7+•** | 0.845 | 0.892 | 0.047 | **A18a+•** | 0.917 | 0.928 | 0.011 |
| **A9+•** | 0.916 | 0.928 | 0.012 | **A18b+•** | 0.912 | 0.925 | 0.013 |
| **A13a+•** | 0.874 | 0.912 | 0.038 | **A20a+•** | 0.858 | 0.901 | 0.043 |
| **A13b+•** | 0.878 | 0.911 | 0.033 | **A20b+•** | 0.867 | 0.902 | 0.035 |
| **A1-•** | 0.777 | 0.849 | 0.072 | **A10a-•** | 0.619 | 0.656 | 0.037 |
| **A2-•** | 0.646 | 0.680 | 0.034 | **A10b-•** | 0.639 | 0.661 | 0.022 |
| **A3-•** | 0.818 | 0.843 | 0.025 | **A14a-•** | 0.556 | 0.601 | 0.045 |
| **A4-•** | 0.560 | 0.606 | 0.046 | **A14b-•** | 0.576 | 0.607 | 0.031 |
| **A5-•** | 0.610 | 0.635 | 0.025 | **A17a-•** | 0.515 | 0.577 | 0.062 |
| **A6-•** | 0.545 | 0.572 | 0.027 | **A19a-•** | 0.543 | 0.587 | 0.044 |
| **A7-•** | 0.763 | 0.854 | 0.091 | **A19b-•** | 0.542 | 0.588 | 0.046 |
| **A8-•** | 0.638 | 0.669 | 0.031 | **A23a-•** | 0.598 | 0.661 | 0.063 |
| **A9-•** | 0.839 | 0.874 | 0.035 | **A23b-•** | 0.594 | 0.662 | 0.068 |

a Data taken from ref. [12]

b Δ = HOMED11(water) – HOMED11(gas)

**Table S5**  Variations of the HOMED indices (δHOMED)a and the relative energies (δ*E*)b for the neutral amine tautomers of adenine when going from purine to adenine

(a) gas phase {DFT(B3LYP)/6-311+G(d,p)}

|  |  | δHOMEDc |  |  |
| --- | --- | --- | --- | --- |
| Tautomer | 5 Bonds | 6 Bonds | 10 Bonds | δ*E*d |
| **A1** | 0.055 | 0.009 | 0.010 | 5.6 |
| **A2** | 0.011 | 0.042 | 0.020 | 0.5 |
| **A3** | 0.053 | 0.021 | 0.025 | -1.8 |
| **A4** | -0.020 | 0.070 | 0.027 | -4.5 |
| **A5** | 0.035 | 0.151 | 0.119 | -10.1 |
| **A6** | -0.020 | 0.037 | -0.038 | 19.1 |
| **A7** | -0.001 | 0.003 | -0.002 | 4.3 |
| **A8** | -0.028 | 0.066 | 0.039 | -6.1 |
| **A9** | 0.011 | 0.001 | 0.004 | 0.0 |

(b) water solution {PCM(water)//DFT(B3LYP)/6-311+G(d,p)}

|  |  | δHOMED |  |  |
| --- | --- | --- | --- | --- |
| Tautomer | 5 Bonds | 6 Bonds | 10 Bonds | δ*E*d |
| **A1** | 0.034 | -0.004 | -0.001 | 1.9 |
| **A2** | 0.012 | 0.042 | 0.023 | 0.3 |
| **A3** | 0.028 | 0.001 | 0.005 | -2.2 |
| **A4** | -0.039 | 0.063 | 0.019 | -7.0 |
| **A5** | 0.011 | 0.125 | 0.098 | -13.2 |
| **A6** | -0.012 | -0.018 | -0.019 | 17.9 |
| **A7** | -0.002 | 0.000 | 0.027 | 1.8 |
| **A8** | -0.040 | 0.066 | 0.024 | -9.1 |
| **A9** | -0.018 | 0.002 | 0.008 | 0.0 |

a δHOMED = HOMED(adenine) - HOMED(purine)

b δ*E* = Δ*E*(adenine) - Δ*E*(purine) in kcal mol^-1^

c Data taken from ref. [12]

d Data for adenine taken from ref. [15] and for purine from ref. [25]

**Table S6**  Variations of the HOMED indices (δHOMED)a and the relative energies (δ*E*)b for the imine tautomers of adenine when going from the structure **a** to **b** (see Fig. S1)

(a) gas phase {DFT(B3LYP)/6-311+G(d,p)}

|  | δHOMEDc | | | | |  |
| --- | --- | --- | --- | --- | --- | --- |
| Tautomer | 5 Bonds | 6 Bonds | 7 Bonds | 10 Bonds | 11 Bonds | δ*E*d |
| **A10** | 0.011 | 0.048 | 0.039 | 0.026 | 0.023 | -4.8 |
| **A11** | 0.009 | 0.063 | 0.051 | 0.038 | 0.033 | -5.3 |
| **A12** | 0.041 | 0.000 | 0.008 | 0.014 | 0.012 | -7.0 |
| **A13** | -0.015 | 0.032 | 0.027 | 0.009 | 0.010 | 0.3 |
| **A14** | 0.085 | 0.059 | 0.047 | 0.010 | 0.009 | -6.4 |
| **A15** | -0.002 | 0.050 | 0.041 | 0.030 | 0.026 | -6.7 |
| **A16** | 0.011 | 0.022 | 0.022 | 0.010 | 0.011 | 1.7 |
| **A17** | 0.044 | -0.117 | -0.091 | -0.055 | -0.045 | -0.5 |
| **A18** | -0.038 | -0.021 | -0.012 | -0.021 | -0.015 | 7.3 |
| **A19** | 0.003 | 0.010 | 0.010 | 0.003 | 0.004 | 0.6 |
| **A20** | 0.001 | 0.005 | 0.006 | 0.004 | 0.005 | 0.2 |
| **A21** | -0.102 | -0.115 | -0.092 | -0.111 | -0.096 | 6.5 |
| **A22** | 0.025 | -0.023 | -0.016 | -0.009 | -0.007 | 0.9 |
| **A23** | -0.003 | -0.011 | -0.006 | -0.013 | -0.009 | 2.1 |

(b) water solution {PCM(water)//DFT(B3LYP)/6-311+G(d,p)}

|  |  |  | δHOMED |  |  |  |
| --- | --- | --- | --- | --- | --- | --- |
| Tautomer | 5 Bonds | 6 Bonds | 7 Bonds | 10 Bonds | 11 Bonds | δ*E*d |
| **A10** | 0.002 | 0.080 | 0.017 | 0.010 | 0.009 | 0.6 |
| **A11** | -0.003 | -0.020 | 0.014 | 0.007 | 0.006 | -0.7 |
| **A12** | 0.013 | 0.014 | 0.011 | 0.013 | 0.011 | -1.6 |
| **A13** | -0.002 | 0.012 | 0.015 | -0.007 | 0.006 | -0.1 |
| **A14** | -0.003 | 0.019 | 0.015 | 0.008 | 0.007 | -1.1 |
| **A15** | 0.001 | 0.020 | 0.016 | 0.013 | 0.011 | -1.5 |
| **A16** | -0.001 | 0.006 | 0.009 | 0.001 | 0.002 | 0.7 |
| **A17** | 0.016 | -0.005 | -0.003 | 0.002 | 0.002 | 0.0 |
| **A18** | -0.004 | -0.002 | 0.001 | -0.004 | -0.001 | 1.6 |
| **A19** | 0.000 | 0.012 | 0.011 | 0.005 | 0.006 | 0.5 |
| **A20** | 0.001 | 0.008 | 0.008 | 0.005 | 0.005 | 0.0 |
| **A21** | -0.014 | -0.038 | -0.029 | -0.028 | -0.016 | 1.5 |
| **A22** | 0.016 | -0.006 | -0.004 | 0.001 | 0.002 | 0.2 |
| **A23** | 0.000 | -0.002 | -0.011 | -0.004 | -0.002 | 0.8 |

a δHOMED = HOMED(**b**) - HOMED(**a**)

b δ*E* = Δ*E*(**b**) - Δ*E*(**a**) in kcal mol^-1^

c Data taken from ref. [12]

d Data taken from ref. [15]

**Figure S2**  Correlations between the total HOMED values estimated in the gas phase and in water solution for the neutral and ionized isomers of purine and adenine

(a) purine

(b) adenine

**Figure S3**  Plots between the relative energies (Δ*E* in kcal mol^-1^) calculated in the gas phase and in water solution for isomers of neutral purine and adenine

(a) purine

(b) adenine

**Figure S4**  Plots of the partial HOMED indices against the relative energies (Δ*E* in kcal mol^-1^) estimated in water solution for the imidazole, pyrimidine, 4-aminopyrimidine, and purine fragments of neutral adenine isomers

(a) imidazole fragment

(b) pyrimidine fragment

(c) 4-aminopyrimidine fragment

(d) purine fragment
